# Fabrication of Silk Scaffold Containing Simvastatin-Loaded Silk Fibroin Nanoparticles for Regenerating Bone Defects

**DOI:** 10.52547/ibj.26.2.116

**Published:** 2021-12-08

**Authors:** Fatemeh Mottaghitalab, Hamidreza Motasadizadeh, Mohammad Ali Shokrgozar, Shahrokh Shojaei, Mehdi Farokhi

**Affiliations:** 1 Nanotechnology Research Centre, Faculty of Pharmacy, Tehran University of Medical Sciences, Tehran, Iran;; 2 Department of Pharmaceutical Nanotechnology, Faculty of Pharmacy, Tehran University of Medical Sciences, Tehran 14174, Iran;; 3 National Cell Bank of Iran, Pasteur Institute of Iran, Tehran, Iran;; 4 Department of Biomedical Engineering Islamic Azad University Central Tehran Branch Tehran 1316943551, Iran

## Abstract

**Background:**

In the present study, a tissue engineered SF scaffold containing simvastatin-loaded SFNPs were used to stimulate the regeneration of the defected bone.

**Methods:**

At first, the porous SF scaffold was prepared using freeze-drying. Then simvastatin-loaded SFNPs were made by dissolvation method and embedded in the SF scaffold. Afterwards, the scaffold and the NPs were characterized in terms of physicochemical properties and the ability to release the simvastatin small molecule.

**Results:**

The results exhibited that the SF scaffold had a porous structure suitable for releasing the small molecule and inducing the proliferation and attachment of osteoblast cells. SFNPs containing simvastatin had spherical morphology and were 174 ± 4 nm in size with -24.5 zeta potential. Simvastatin was also successfully encapsulated within the SFNPs with 68% encapsulation efficiency. Moreover, the small molecule revealed a sustained release profile from the NPs during 35 days. The results obtained from the *in vitro* cell-based studies indicated that simvastatin-loaded SFNPs embedded in the scaffold had acceptable capacity to promote the proliferation and ALP production of osteoblast cells while inducing osteogenic matrix precipitation.

**Conclusion:**

The SF scaffold containing simvastatin-loaded SFNPs could have a good potential to be used as a bone tissue-engineered construct.

## INTRODUCTION

Bone tissue plays important roles in the human body, such as protecting the vital organs, blood production, preserving minerals, homeostasis, regulating blood pH, and producing mesenchymal stem cells and hematopoietic stems cell, and so forth. Bone defects are generally produced upon surgery, trauma, osteomyelitis, osteoarthritis, and bone fractures^[^^1^^]^. More than 450,000 individuals suffer from bone defects in U.S. annually^[^^1^^]^. 

Autologous grafts are the gold standards for treating bone injuries. However, the need for a second surgery, tissue removal, and restricted donor site limit the application of this type of strategy. Consequently, it is reasonable to use tissue engineering strategies as a potential alternative for autologous grafts to regenerate bone defects. A bone tissue engineered construct is usually composed of a biocompatible and biodegradable polymeric matrix containing various growth factors^[^^2^^,^^3^^]^. Although growth factors are desirable components to induce osteogenesis, the high cost, low stability, low half-life, unwanted immunogenicity etc. restrict their usage in tissue engineering applications. It is assumed that small molecules could be potential alternatives for growth regeneration, cost-effectiveness, and higher stability. Small molecules are organic components with a molecular weight of less than 1,000 Dalton. The small size of these molecules facilitates their transit through the cell membrane phospholipid bilayer and triggers the signaling pathways inside the cell. The osteogenic small molecules are a group of materials that provoke the differentiation of multipotent mesenchymal stem cells into adult osteoblast cells^[^^4^^-^^7^^]^. 

Recently, various osteogenic small molecules, such as dexamethasone, phenamil, oxysterol, purmor-phamine, fingolimod (FTY720), statin family, and bisphosphonates, have been used in bone tissue engineered constructs^[^^6^^,^^7^^]^. The small molecules in the statin family are potential inhibitors that are widely used for treating hypercholesterolemia. Moreover, these molecules are capable to induce bone regeneration by stimulating the osteoblast cells and bone stromal cells through bone morphogenic protein signaling pathways. The slow release rate of statins from drug delivery systems also made them desirable molecules for treating osteoporosis. Simvastatin, lovastatin, atrovastattin, and rosuvastatin are some of the small molecules in the statin family and are capable of promoting osteogenesis^[^^8^^-^^10^^]^. To inhibit the off-target properties of statin on undesired tissues, it can be loaded in the targeted drug delivery carriers with the ability to release the cargo in the site of action. In recent years, various carriers, including microspheres, nanaospheres, nanocapsules, nanofibers, and so on, have been used for small molecule delivery^[^^8^^,^^11^^,^^12^^]^. Among these systems, the nanoparticulate platforms have gained more attention due to higher stability, high surface to volume ratio, tunable size, and longer half-life. The polymeric NPs, whether synthetic or natural, are good candidates for small molecule delivery. However, it is necessary to choose a suitable polymeric construct according to the delivered molecule in terms of biocompatibility, biodegradability, solubility, controlled release behavior, mechanical strength, and chemical properties. Among different polymeric NPs, those synthesized from natural polymers are more utilized for bone regeneration due to less immunogenicity, acceptable biodegradability, and tunable processability^[^^13^^]^. In the present study, we used SF, as a potential natural polymer, for fabricating the bone scaffold^[^^14^^-^^16^^]^. To increase the ability of this scaffold for bone regeneration, we embedded the SFNP-containing simvastatin small molecules within the SF scaffold. It was hypothesized that this construct with great biocompatibility and biodegradability, high capacity for drug loading, good performance for cell endocytosis, controlled release behavior, and high half-life in blood circulation could enhance the proliferation and attachment of osteoblast cells *in vitro*^[^^17^^,^^18^^]^.

Herein, after the preparation of SF scaffold containing simvastatin-loaded SFNPs, the structure was characterized in terms of physico-chemical properties. The release profiles of simvastatin from both scaffold and SFNPs were also examined. Finally, the capability of the scaffold in provoking the osteoblast cells activities, such as proliferation, attachment, alkaline phosphate production, and matrix precipitation, were evaluated, as well. 

## MATERIALS AND METHODS

**Materials**

Sodium carbonate, lithium bromide, MTT, methanol, isopropanol, paraformaldehyde, simvastatin, and dialysis tube (cut-off 12 kDa) were purchased from Sigma-Aldrich (USA). DMEM, fetal bovine serum, and PBS were prepared from Gibco (USA). Rabbit osteoblast cells were obtained from the National Cell Bank of Iran (Pasteur Institute of Iran, Tehran).

**Preparation of SFNPs**

Prior to SFNP preparation, the SF was extracted from* Bombyx mori* silkworm silk fiber. For this purpose, the fibers were firstly dissolved in 0.02 M sodium carbonate for two consecutive 30 min, washed with PBS for three times, and dried at room temperature for 24 h. Secondly, the degummed fibers were dissolved in 9.3 M of lithium bromide at 60 °C for 4 h and then dialyzed using a dialysis tube against water for three days. At last, three types of SFNPs were prepared by adding ethanol, as a dissolving agent, to 2% SF solution. The NPs were then centrifuged at 35000 ×g for 10 min, and the participated NPs were freeze-dried for further use. The size and zeta potential of the prepared NPs were characterized using dynamic light scattering (Malvern, UK). The morphology of SFNPs was also analyzed by field emission scanning electron microscopy.

**Drug loading efficacy**

To encapsulate simvastatin into SFNPs, different concentrations of simvastatin (1, 2, and 3 mg/mL) and a constant concentration of SFNPs (1%) were considered. For this purpose, the small molecule was dissolved in 1 mL of deionized water and then added to the SFNPs solution. The drug loading, encapsulation efficiency, and production yield were measured at 238 nm by spectroscopy using the following calculations: 

$\mathrm{Encapsulation efficiency}\left(\%\right)$ = 

$\frac{\mathrm{total amount of drug}–\mathrm{amount of unbounded drug}}{\mathrm{total amount of drug}}\times 100$

$\mathrm{Loading capacity}\left(\%\right)=$

$\frac{\mathrm{total amount of drug}–\mathrm{amount of unbounded drug}}{\mathrm{weight of NPs}}=\times 100$

Moreover, to confirm the interaction between simvastatin and SFNPs, FTIR was performed at 400-4000 cm^-1^ wavelength. 

**Drug release study**

The freeze-dried SFNPs were dissolved in 1 mL of PBS and transferred to a dialysis tube, immersed in 2 mL of PBS solution with pH 7.4 and kept at 37 °C. In each interval, the release medium was replaced with an equal amount of fresh PBS, and the cumulative release rate of simvastatin was measured at 238 nm using spectrophotometry. 

**Preparation of SF scaffold containing simvastatin/ SFNPs**

To fabricate SF scaffold containing simvastatin/ SFNPs, 5 mg of the NPs were added and dispersed in 1 mL of degummed SF solution. The solution was then transferrred to a 24-well culture plate and preserved at -70 °C overnight. Afterwards, freeze-drying was applied for 48 h to fabricate the porous SF scaffold. Subsequently, the freeze-dried scaffold was crosslinked by immersion in methanol for two consecutive 10 min. The scaffold was then washed three times with PBS. The mechanical strength of the scaffold was examined in the pressure mode, triplicate. To assess the porosity of the scaffold, the freeze-dried SF scaffold was first immersed in ethanol and then removed after 10 min. Subsequently, the porosity percentage was measured according to the following formula:

$\mathrm{Porosity}\left(\%\right)=\frac{V_{1}-V_{3}}{V_{2}-V_{3}}$

Where V_1_ is the initial volume of ethanol, V_2_ is the volume of the ethanol after immersing the scaffold, and V_3_ is the residual volume of the ethanol after removing the scaffold from the solution.

**Scaffold biocompatibility**

The biocompatibility of the scaffold was evaluated using an indirect test according to ISO 10993-5 standard. To this end, the scaffolds with an identified surface area were immersed in 1 mL of culture medium and kept for cell viability test for 3, 7, and 14 days. Then rabbit osteoblast cells were cultured in a 24-well culture plate at 37 °C and 5% CO_2_. After 24 h, the culture media was replaced with the extraction solutions of the scaffold and kept at 37 °C for another 24 h. The day after, the whole media was replaced with 0.5 mg/mL of MTT solution and placed in an incubator for 4 h. Finally, the participated formazan was dissolved by isopropanol, and the absorbance was read at 570 nm using an ELISA reader. Three groups were considered for MTT assay: (1) TPS, (2) bulk SF scaffold, and (3) SF scaffold containing simvastatin/ SFNPs. 

**ALP activity**

The rate of ALP production by the osteoblast cells was measured by a commercial ALP kit (Pars Azmoon, Iran) according to manufacturer's protocol. Briefly, about 5,000 osteoblast cells were cultured in a 24-well culture plate for 24 h. The culture medium was replaced with the extraction solutions of the scaffolds and kept in an incubator for three days. Then the supernatant of the rabbit osteoblast cells (containing 10 μL of cells) was added to the ALP kit reagent (1,000 μL), and the absorbance was read at 405 nm using an ELISA reader (Epoch 2 microplate spectrophotometer, USA). 

**Alizarin red staining**

To confirm the ability of rabbit osteoblast cells for mineralization and matrix calcification next to the SF scaffold, Alizarin red staining was performed. Firstly, 5,000 osteoblast cells were cultured in a 24-well culture plate. After 24 h, the culture medium was replaced with the extraction solutions of the scaffold for three days. Afterwards, the cells were washed with 0.1 M of NaCl and fixed in 1% paraformaldehyde (4 v/v%) for 30 min and stained with Alizarin red at room temperature. The stained regions were evaluated using light microscopy, and the results were reported, qualitatively. 

**Fig. 1:**
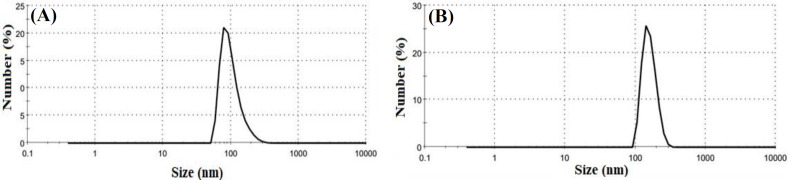
Size distribution of SFNPs before (A) and after (B) drug loading. The SFNPs had uniform size distribution

**Statistical analysis**

Statistical analysis was performed by using one-way ANOVA with SPSS 16.0 (SPSS, USA). *p* values less than 0.05 were considered statistically significant.

## RESULTS AND DISCUSSION

**Size and zeta potential of SFNPs**

**Fig. 2:**
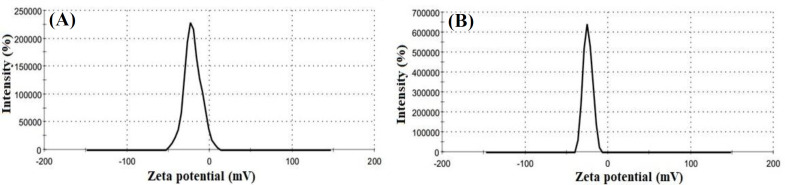
Zeta potential of SFNPs before (A) and after (B) drug loading

Based on the dynamic light scattering data, the size of SFNPs was 146 nm with PDI 0.2 (Fig. 1A) before drug loading, which increased to 171 nm (Fig. 1B) thereafter. The mild increase in the size of the SFNPs might be related to locating of hydrophobic simvastatin in the core of SFNPs, which induced the accumulation of polymeric chains on the surface of the SFNPs. Moreover, the surface charge of SFNPs was changed from -20.3 mV to -24.4 mV after drug loading (Fig. 2A and 2B). This behavior was due to the existence of amino acids with a negative charge in the structure of SFNPs; the increase in the negative charge after loading simvastatin might be also related to polymeric chain accumulation on the surface of SFNPs.

**Surface morphology of SFNPs**

**Fig. 3:**
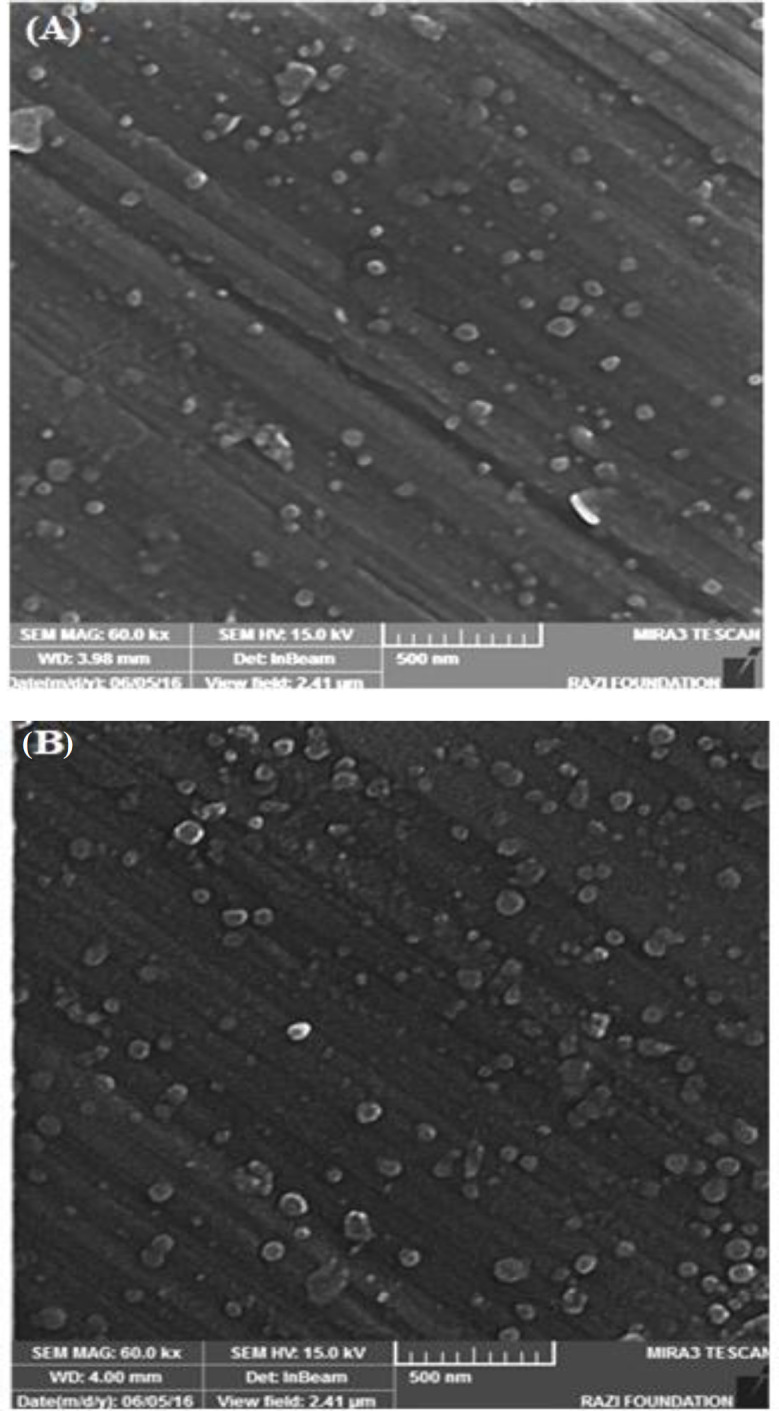
SEM images of SFNPs before (A) and after (B) drug loading. The NPs had spherical morphology with uniform size distribution (magnification: 500 nm)

SEM micrographs showed that SFNPs had spherical morphology without accumulation due to the repulsion of negative charges at the surface of NPs. This phenomenon resulted in the uniform distribution and stability of SFNPs. As can be seen in Figure 3, the size distribution of SFNPs has slightly increased after loading simvastatin. 

**Structural analysis of SFNPs**

**Fig. 4:**
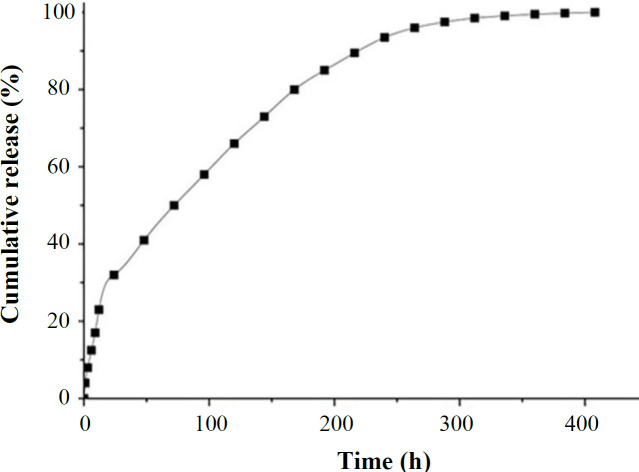
FTIR spectra of bulk simvastatin, SFNPs, and simvastatin-loaded SFNPs. No specific changes were observed after loading the drug on SFNPs.Sim, simvastatin

In the FTIR spectra, the characteristic peaks of SFNPs related to amine I, amine II, amine III residues were present at 1230 cm^-1^, 1518 cm^-1^, and 1630 cm^-1^, respectively (Fig. 4). Moreover, simvastatin revealed specific peaks at 3552 cm^-1^ and 3749 cm^-1^ (O−H stretch vibration), as well as at 1730 cm^-1^, and 1164 cm^-1^ and 1066 cm^-1^ (stretch vibration of −C−O and −C=O carbonyl functional group). However, after loading simvastatin on SFNPs, no significant changes were observed in the FITR spectra of SFNPs because most of the drug molecules were encapsulated within the NPs and a slight portion of the drug was loaded only on the SFNPs (Fig. 4).

**Drug loading on SFNPs**

 By increasing the drug concentration from 1 mg/mL to 2 mg/mL, the drug loading percentage and encapsulation efficiency increased. However, the samples containing 3 mg/mL of simvastatin showed significantly lower drug loading percentage and encapsulation efficiency.

**Simvastatin release study**

**Fig. 5:**
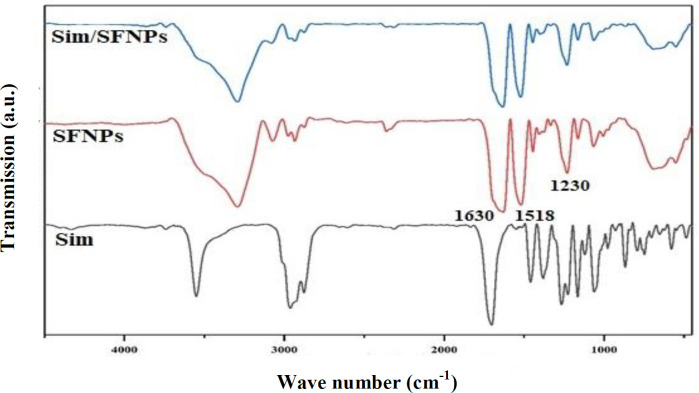
Release profile of simvastatin from SFNPs at pH 7.4. The drug had a slow release rate from the NPs due to strong hydrophobic interactions between the drug and SFNPs

 The release profile of simvastatin at pH 7.4 is shown in Figure 5. The data revealed that simvastatin had a slow release rate from SFNPs; the release of the drug had a sustained kinetics from day one to eight and reached the plateau thereafter. During 15 days, nearly 100% of the drug was released from the NPs. The sustain release rate of simvastatin might be related to strong hydrophobic interactions between the drug and hydrophobic block of SFNPs.

**Fig. 6:**
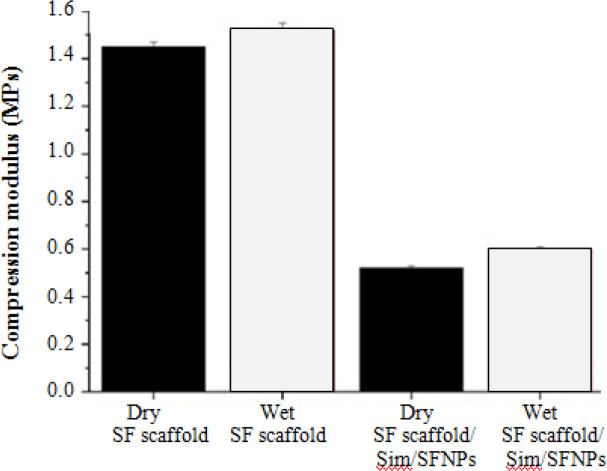
Compression modulus of dry and wet SF scaffolds before and after adding SFNPs. As a reinforcement, SFNPs slightly increased the compression modulus of bulk SF scaffolds (*p *< 0.05)

**Mechanical properties of the scaffold**

Figure 6 shows that by increasing the ratio of SFNPs within the scaffold, the mechanical strength of the scaffold enhances because the NPs acted as a reinforcement. Based on the obtained results, the compression moduli of dry SF scaffolds and wet SF scaffolds without SFNPs were 1.45 MPa and 1.52 

**Fig. 7:**
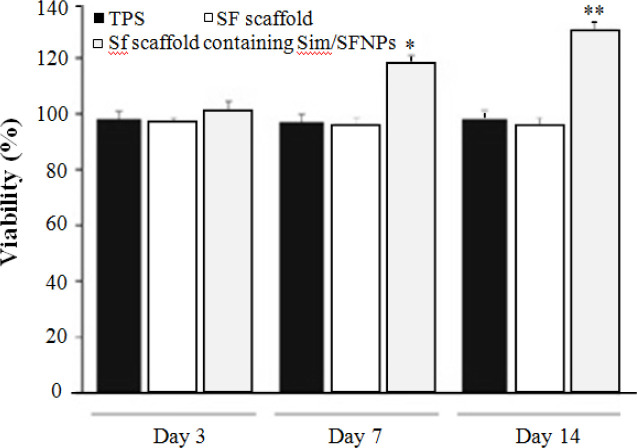
MTT results showing increased proliferation rate of osteoblast cells from day 3 to day 14. ^*^ and ^** ^ indicate significant increase in the proliferation rate of the cells on SF scaffolds containing simvastatin/SFNPs compared to the control on days three and seven, respectively. Sim, simvastatin

MPa, respectively. However, after adding 5 mg SFNPs to the mitochondrial function of cells. However, it was reported that simvastatin induces negative effects on the proliferation and mineralization of human primary osteoblasts^[^^20^^]^.

**ALP assay**

**Fig. 8:**
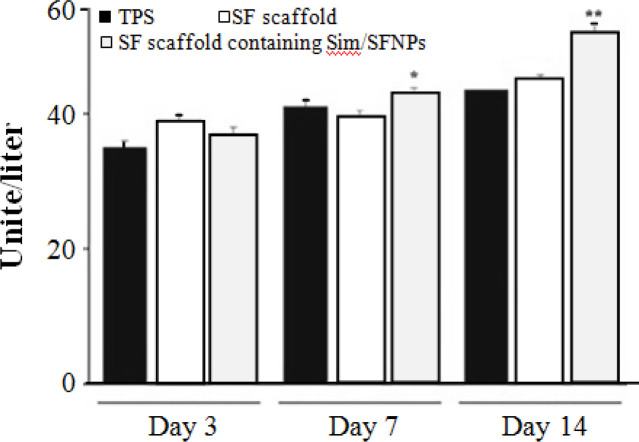
ALP production of osteoblast cells from day 3 to day 14. ^*^ and ^**^ indicate significant increase in the proliferation rate of the cells on SF scaffolds containing simvastatin/SFNPs compared with the control on days three and seven, respectively. Sim, simvastatin

 The rate of ALP production from osteoblast cells indicated no significant differences between the groups after three days; however, the ALP production of osteoblast cells significantly increased on day 7 and 14 next to SF scaffolds containing simvastatin/SFNPs compared to bulk SF scaffolds and TPS (*p *< 0.05). Moreover, the ALP production on day 14 was more than days three and day seven (Fig. 8). The results obtained from ALP assay confirmed those achieved from MTT assay. A study showed that the released simvastatin from an injectable scaffold promoted ALP activity and mineral matrix deposition^[^^21^^]^. Further, it was demonstrated that gelatin-nanofibrillar cellulose-β to the mitochondrial function of cells. However, it was within the SF scaffolds, the compression moduli of the dry and wet scaffolds increased to 1.54 MPa and 0.6 MPa. Nonetheless, no significant differences were observed between the mechanical properties of the scaffolds before and after adding the SFNPs.

**Porosity measurement of SF scaffold**

The results obtained from porosity measurement exhibited that the porosity of SF scaffolds increased from 66.73% to 71.3% after adding 5 mg of SFNPs. It is assumed that SFNPs could avoid the scaffold from shrinkage and thus increase the porosity. However, a mild shrinkage was observed in those scaffolds without NPs, which reduced the porosity percentage.

**Fig. 9:**
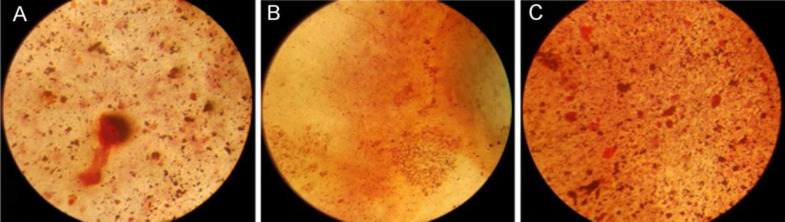
Alizarin staining of osteoblast cells cultured on (A) TPS, (B) bulk SF scaffold, and (C) SF scaffold containing simvastatin/SFNPs

**Biocompatibility assay**

The MTT assay results revealed acceptable bio-compatibility of SF scaffolds, whether with or without SFNPs, compared to the control group. Figure 7 shows that the proliferation rate of osteoblast cells on bulk SF scaffold, SF scaffold containing simvastatin-loaded SFNPs, and TPS (control group) had no significant differences on day three. However, on day seven, osteoblast cells exhibited significantly higher proliferation rate on SF scaffolds containing simvastatin-loaded SFNPs in comparison to bulk SF scaffolds and TPS (*p *< 0.05). This trend was also observed on day 14 (Fig. 8). Similarly, Chuang *et al.*^[^^19^^] ^found that simvastatin significantly enhanced the proliferation of human osteoblasts, which may related tricalcium phosphate hydrogel containing simvastatin stimulated specific bone gene expression, e.g. osteopontin, osteocalcin, and ALP. These data also showed that hydrogel, along with its osteoconductive architecture, releases the optimum concentration of simvastatin to stimulate osteogenesis^[^^22^^]^. Similarly, the mRNA expression of osteoblast-related genes such as collagen type I alpha 1, ALP, osteopontin, osteocalcin, and vascular endothelial growth factor A improved in human adipose tissue-derived mesenchymal stem cells cultured on titanium dioxide scaffold coated with alginate hydrogels loaded with simvastatin compared to scaffolds without simvastatin^[^^23^^]^.

**Bone Matrix mineral staining**

 The Alizarin red staining exhibited that the osteoblast cells cultured on SF scaffolds containing simvastatin/SFNPs induced more osteogenesis compared to those cells cultured on bulk SF scaffolds and TPS (Fig. 9). It has been reported that simvastatin is able to promote osteogenic differentiation of mesenchymal stem cells *in* *vitro*. Moreover, the local release of simvastatin from calcium sulfate scaffolds enhances bone regeneration, and its effects is equal to that of recombinant human bone morphogenetic protein-2^[^^24^^]^.

## DECLARATIONS

### Ethical statement

Not applicable. 

### Data availability

Data supporting this article are included within the article and are available from the corresponding author on reasonable request.

### Author contributions

Fatemeh Mottaghitalab: Advisor; Hamidreza Motasadizadeh: Running the project; Mohammad Ali Shokrgozar: Project management and supervisor; Shahrokh Shojaei: Advisor; Mehdi Farokhi: Supervisor

### Conflict of interest

None declared.

### Funding/support

This work has financially been supported by the National Institute for Medical Research Development, Tehran, Iran (grant no. 958833).
